# Linear Characteristics of the Differences in Phase Tangents of Triple-Coil Electromagnetic Sensors and Their Application in Nonmagnetic Metal Classification

**DOI:** 10.3390/s22197511

**Published:** 2022-10-03

**Authors:** Dong Wang, Zhijie Zhang, Wuliang Yin, Haoze Chen, Huidong Ma, Guangyu Zhou, Yuchen Zhang

**Affiliations:** 1 School of Instrument and Electronics, North University of China, Taiyuan 030051, China; 2 School of Electrical and Electronic Engineering, University of Manchester, Manchester M60 1QD, UK

**Keywords:** eddy current testing, lift-off, metal classification, phase tangent, triple-coil sensor

## Abstract

Metal sorting is the first step in scrap metal recycling. The traditional magnetic separation method can classify ferromagnetic metals, but it is not applicable to some nonmagnetic metals with higher value. To address this situation, we propose an eddy current testing (ECT) technology-based method for classifying nonmagnetic metals. In this study, a triple-coil electromagnetic sensor, which works as two coil pairs, is tested. By analyzing the physical model of the sensor, a feature related to the conductivity of the sample under test is obtained as the difference in the tangent of the impedance changes in the two coil pairs. Additionally, we derive a linear relationship between this feature and the lift-off height, which is verified experimentally and will help to solve the classification error caused by the variation in the lift-off height. In addition, we find that the excitation frequency does not affect this linear feature. Moreover, in this study, the spectrum scanning method is converted into a single-frequency measurement, and the time consumption is greatly reduced, which improves the efficiency of the real-time metal classification system.

## 1. Introduction

The introduction of the circular economy (CE) concept has led to sufficient attention being paid to the metal recycling industry [1,2]. With this came the demand for greater accuracy, speed and cost effectiveness in the sorting and recycling of scrap metals [3]. Electrical conductivity, as a fundamental property of metals, is very suitable on a classification basis for the sorting and recycling of scrap metals [4,5].

Eddy current testing (ECT) has performed well in the field of non-destructive testing (NDT), such as in crack detection [6], thickness measurement [7,8,9], metal magnetic and electrical conductivity measurement [10,11], and is considered one of the most promising methods due to its advantages of being non-contact, reliable, and fast. To address the issue of electrical conductivity measurement, researchers have explored various excitation methods [12,13,14,15], such as feature extraction [16], sensor design [17,18], and compensation algorithm derivation [19]. Among them, the single-frequency excitation method is the most suitable for real-time conductivity classification systems due to its ability to produce data in a timely manner.

In previous studies, different features of inductance change with conductivity as a variable have been extracted as the basis for nonmagnetic metal classification. Additionally, various compensation algorithms have been designed to eliminate the negative effect on the classification accuracy caused by the lift-off (distance from the bottom of the sensor to the top of the sample under test) [20]. Du et al. achieved the classification of tilted metals by fitting a linear relationship between the tilt angle and phase using the phase of inductance change as a feature combined with the tilt angle obtained from the photoelectric sensor [21]. Then, they proposed a circle-fitting method to extract the global characteristics of different mutual metal inductance trajectories on the complex plane, so that metals with larger tilt angles could also be accurately classified [22]; a pseudo-linear relationship between the magnitude–phase curve of inductance change was also found [23]. Liu et al. extracted the characteristic slope from the normalized inductance trajectory on a complex plane and used the least squares method to classify metals with a larger tilt angle [24]. Wang et al. found a linear relationship between the logarithm of the phase of the impedance change and the electrical conductivity of the metal and considered the effects of the excitation frequency, lift-off, and relative permeability [25]. Lu et al. derived a compensation algorithm to compensate for the peak frequency deviation caused by the lift-off, which was experimentally verified [19]. Yin et al. designed a new triple-coil electromagnetic sensor that uses the difference in the peak frequency of the impedance change between two coil pairs to measure the plate thickness, which is virtually immune to lift-off variations [26].

This paper first describes the structure and parameters of the triple-coil electromagnetic sensor used. Next, a theoretical analysis of the sensor model is performed to obtain the feature for classification. The effect of the lift-off height on the feature is also considered, and it is found that there is a linear relationship between the feature and the lift-off height. Then, four nonmagnetic metals are used to experimentally verify the feasibility of using this feature for classification, and the linear relationship is used to eliminate the negative effect of lift-off height on the classification. Finally, the accuracy of this classification method is verified.

## 2. Sensor Configuration

**Figure 1:**
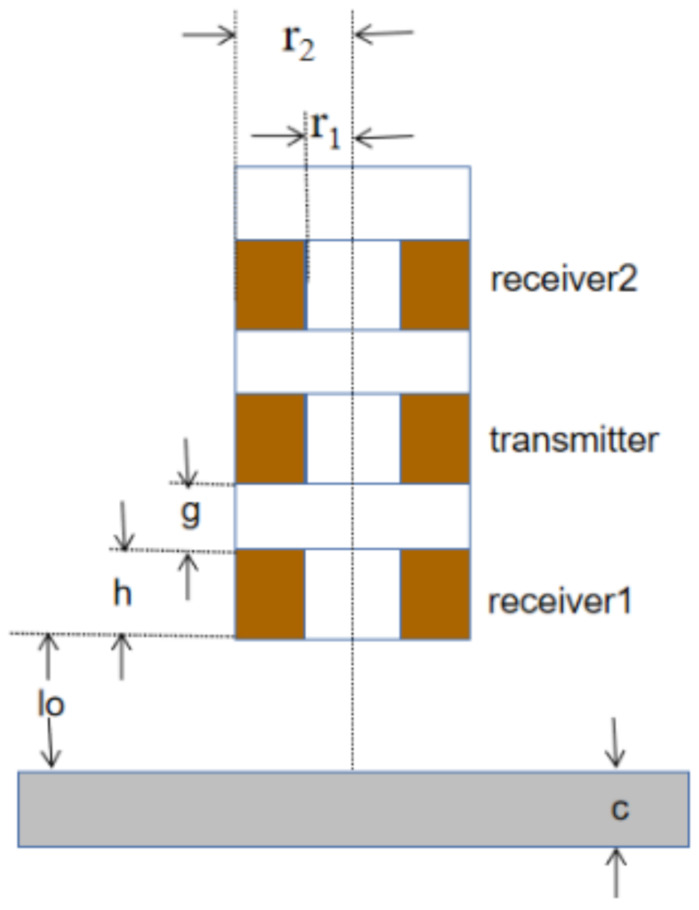
Sensor structure.

**Figure 2:**
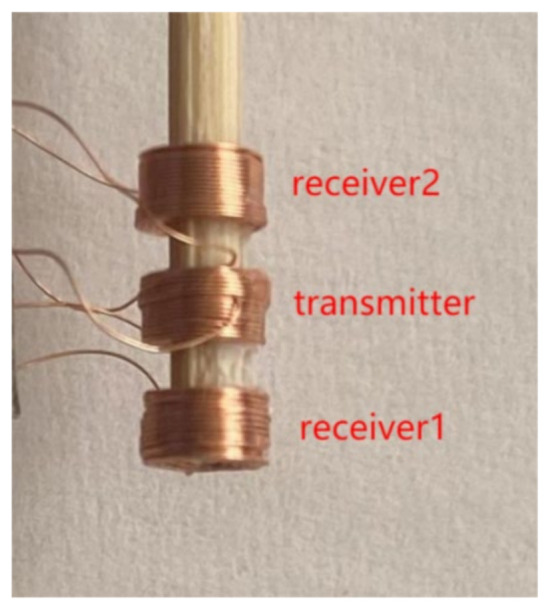
Physical picture of the sensor.

The diagram of the triple-coil electromagnetic sensor is shown in Figure 1. The sensor consists of three coils of the same size, co-axially arranged and equally spaced, the lower coil being receiver 1, the middle coil being the transmitter, and the upper coil being receiver 2. These three coils operate as two coil pairs, where the transmitter and receiver 1 coils compose the first coil pair (TR1), and the transmitter and receiver 2 coils compose the second coil pair (TR2). The distance between the bottom of the receiver 1 coil and the top surface of the sample is the lift-off (*l*_o_). Due to the fixed structure of the sensor, the difference in the lift-off of the two coil pairs is constant. A physical picture of the sensor is provided in Figure 2, and the parameters are shown in Table 1.

## 3. Theoretical Derivation

In this section, a feature with magnetic permeability and electrical conductivity as its variables is obtained through mathematical derivation, and the effect of lift-off height on this feature is also considered.

### 3.1. Derivation of the Classification Feature

Starting with the analytical model of Dodd and Deeds [27], the distribution of the vector potential generated by a circular excitation coil on a homogeneous, nonmagnetic infinite half-plane is described, and the inductance change in an air-core coil caused by the plate is given. The difference in the complex inductance is $\Delta L\left(\omega \right)=L\left(\omega \right)-L_{A}\left(\omega \right)$, where $L\left(\omega \right)$ is the coil inductance above a plate, and $L_{A}\left(\omega \right)$ is the inductance in free space. Using the triple-coil sensor described in this paper, $\Delta L_{1}\left(\omega \right)$ and $\Delta L_{2}\left(\omega \right)$ can be obtained, representing the inductance changes in TR1 and TR2, respectively.

When the size of the sample exceeds five times the size of the coil, the sample can be considered as an infinite half-plane. According to the revised Dodd and Deeds formulas described in [26], the inductance changes in two coil pairs, $\Delta L_{1}\left(\omega \right)$ and $\Delta L_{2}\left(\omega \right)$, are as follows:

(1) $$\text{Δ}L_{1}(\omega )=K\int_{0}^{\infty }\frac{P^{2}(\alpha )}{\alpha^{6}}A_{1}(\alpha )\phi (\alpha )d\alpha$$

(2) $$\text{Δ}L_{2}(\omega )=K\int_{0}^{\infty }\frac{P^{2}(\alpha )}{\alpha^{6}}A_{2}(\alpha )\phi (\alpha )d\alpha$$

where

(3) $$\phi (\alpha )=\frac{\left(\alpha_{1}+\mu_{r}\alpha \right)\left(\alpha_{1}-\mu_{r}\alpha \right)-\left(\alpha_{1}+\mu_{r}\alpha \right)\left(\alpha_{1}-\mu_{r}\alpha \right)e^{2\alpha_{1}c}}{-\left(\alpha_{1}-\mu_{r}\alpha \right)\left(\alpha_{1}-\mu_{r}\alpha \right)+\left(\alpha_{1}+\mu_{r}\alpha \right)\left(\alpha_{1}+\mu_{r}\alpha \right)e^{2\alpha_{1}c}}$$

(4) $$\alpha_{1}=\sqrt{\alpha^{2}+j\omega \sigma \mu_{r}\mu_{0}}$$

(5) $$K=\frac{\pi \mu_{0}N^{2}}{h^{2}{\left(r_{1}-r_{2}\right)}^{2}}$$

(6) $$P(\alpha )=\int_{\alpha r_{1}}^{\alpha r_{2}}xJ_{1}(x)dx$$

(7) $$A_{1}(\alpha )=e^{-\alpha (2l_{o}+h+g)}\left(1+e^{-2\alpha h}\right)$$

(8) $$A_{2}(\alpha )=e^{-\alpha (2l_{o}+3h+3g)}\left(1+e^{-2\alpha h}\right)$$

where *ω* is the excitation frequency, *μ*_r_ and *μ*_0_ denote the relative permeability of the sample and vacuum permeability, respectively, and *σ* denotes the conductivity. *N* denotes the number of turns in the coil, *r*_1_ and *r*_2_ denote the inner and outer radius of the coil, *h* is its height, *g* is the gap between the coils, lo is the lift-off, and *c* denotes the thickness of the plate. *J*_1_(*x*) is a first order Bessel function of the first kind; *α* and *x* are the integration variables.

It is stated in [20] that ϕ(α) varies much more slowly compared with the rest of the integration variables, while $\Delta L\left(\omega \right)$ can be approximated by taking ϕ(α) outside the integral and assuming the maximum value at a characteristic spatial frequency $\alpha_{0}$. After the approximation,

(9) $$\Delta L_{1}(\omega )=\phi \left(\alpha_{0}{}_{1}\right)\Delta L_{01}$$

(10) $$\Delta L_{2}(\omega )=\phi \left(\alpha_{0}{}_{2}\right)\Delta L_{02}$$

where

(11) $$\phi \left(\alpha_{01}\right)=\frac{\left(\alpha_{1}+\mu_{r}\alpha_{01}\right)\left(\alpha_{1}-\mu_{r}\alpha_{01}\right)-\left(\alpha_{1}+\mu_{r}\alpha_{01}\right)\left(\alpha_{1}-\mu_{r}\alpha_{01}\right)e^{2\alpha_{1}c}}{-\left(\alpha_{1}-\mu_{r}\alpha_{01}\right)\left(\alpha_{1}-\mu_{r}\alpha_{01}\right)+\left(\alpha_{1}+\mu_{r}\alpha_{01}\right)\left(\alpha_{1}+\mu_{r}\alpha_{01}\right)e^{2\alpha_{1}c}}$$

(12) $$\phi \left(\alpha_{02}\right)=\frac{\left(\alpha_{1}+\mu_{r}\alpha_{02}\right)\left(\alpha_{1}-\mu_{r}\alpha_{02}\right)-\left(\alpha_{1}+\mu_{r}\alpha_{02}\right)\left(\alpha_{1}-\mu_{r}\alpha_{02}\right)e^{2\alpha_{1}c}}{-\left(\alpha_{1}-\mu_{r}\alpha_{02}\right)\left(\alpha_{1}-\mu_{r}\alpha_{02}\right)+\left(\alpha_{1}+\mu_{r}\alpha_{02}\right)\left(\alpha_{1}+\mu_{r}\alpha_{02}\right)e^{2\alpha_{1}c}}$$

(13) $$\Delta L_{01}=K\int \frac{P^{2}(\alpha )}{\alpha^{6}}A_{1}(\alpha )d\alpha$$

(14) $$\Delta L_{02}=K\int \frac{P^{2}(\alpha )}{\alpha^{6}}A_{2}(\alpha )d\alpha$$

Note that in (9) and (10), the phase signature of the inductance change is solely determined by $\phi (\alpha_{01})$ or $\phi (\alpha_{02})$. For nonmagnetic metals, the relative permeability is approximately 1. By substituting $e^{2\alpha_{1}c}$ with $1+2\alpha_{1}c$ in (11) and (12),

(15) $$\phi \left(\alpha_{01}\right)\approx \frac{j\omega \sigma \mu_{r}\mu_{0}c}{j\omega \sigma \mu_{r}\mu_{0}c+2\alpha_{01}^{2}c+2\alpha_{01}+2\alpha_{01}\alpha_{1}c}$$

(16) $$\phi \left(\alpha_{02}\right)\approx \frac{j\omega \sigma \mu_{r}\mu_{0}c}{j\omega \sigma \mu_{r}\mu_{0}c+2\alpha_{02}^{2}c+2\alpha_{02}+2\alpha_{02}\alpha_{1}c}$$

Letting

(17) $$\omega_{1}=\frac{2\alpha_{01}^{2}c+2\alpha_{01}}{\sigma \mu_{r}\mu_{0}c}$$

(18) $$\omega_{2}=\frac{2\alpha_{02}^{2}c+2\alpha_{02}}{\sigma \mu_{r}\mu_{0}c}$$

When the thickness of the plate is much greater than the diameter of the coil, $\alpha_{01}c\gg 1$ (or $\alpha_{02}c\gg 1$), (17) and (18) can be further simplified as

(19) $$\omega_{1}=\frac{2\alpha_{01}^{2}}{\sigma \mu_{r}\mu_{0}}$$

(20) $$\omega_{2}=\frac{2\alpha_{02}^{2}}{\sigma \mu_{r}\mu_{0}}$$

Equations (15) and (16) can be expressed as

(21) $$\phi (\alpha_{01})=\frac{j\omega /\omega_{1}}{j\omega /\omega_{1}+1+2\alpha_{01}\alpha_{1}c/\left(2\alpha_{01}^{2}c+2\alpha_{0}\right)}$$

(22) $$\phi (\alpha_{02})=\frac{j\omega /\omega_{2}}{j\omega /\omega_{2}+1+2\alpha_{02}\alpha_{1}c/\left(2\alpha_{02}^{2}c+2\alpha_{0}\right)}$$

From (21) and (22), we can see that the phase spectrum of inductance change approximates a first-order system, with the imaginary part peaking at frequency *ω*_1_ or *ω*_2_. By combining (21) and (22), the following relationship exists between inductance change and impedance change:

(23) ΔZ(ω)=jω×ΔL(ω)

We then arrive at

(24) tanθ1=Im(ΔZ1)Re(ΔZ1)=−Re(ΔL1)Im(ΔL1)=−ωω1

(25) tanθ2=Im(ΔZ2)Re(ΔZ2)=−Re(ΔL2)Im(ΔL2)=−ωω2

where $\theta_{1}$ and $\theta_{2}$ denote the phase angles of impedance change for TR1 and TR2, respectively. With Equations (24) and (25), we obtain the relationship between the phase tangent of the impedance change, the excitation frequency, and the peak frequency, which means that the peak frequency can be expressed in terms of the phase tangent and the excitation frequency. In this way, the peak frequency obtained by sweeping can be obtained by single-frequency excitation instead, which will greatly reduce the measurement time and improve the classification efficiency.

Then, (19) and (20) are combined with (24) and (25) to obtain a feature with conductivity as a variable:

(26) $$\begin{array}{c} \tan\theta_{1}-\tan\theta_{2}=-\omega *(\frac{1}{\omega_{1}}-\frac{1}{\omega_{2}})\\ =2\omega \sigma \mu_{r}\mu_{0}\frac{\alpha_{01}^{2}-\alpha_{02}^{2}}{\alpha_{01}^{2}\alpha_{02}^{2}} \end{array}$$

### 3.2. Effect of Lift-Off Height on the Feature

In [17], a simple trigonometric function $\sin^{2}\left(\alpha \pi /2\alpha_{01}\right)$ (or $\sin^{2}\left(\alpha \pi /2\alpha_{02}\right)$) is used to approximate the $\left(P^{2}(\alpha )/\alpha^{6})A_{1}(\alpha \right)$ term in Equation (1) (or the $\left(P^{2}(\alpha )/\alpha^{6})A_{2}(\alpha \right)$ term in Equation (2)) and, finally, the shift in $\alpha_{01}$ (or $\alpha_{02}$) caused by the lift-off can be derived. Since the $\left(P^{2}(\alpha )/\alpha^{6}\right)$ term is the main contributor to the integration, not the $A_{1}(\alpha )$ term (or $A_{2}(\alpha )$ term), it is reasonable to assume that $\alpha_{01}$ ≈ $\alpha_{02}$ = $\alpha_{0}$. Additionally, the revised $\alpha_{01r}$ and $\alpha_{01r}$ are

(27) $$\alpha_{01r}=\alpha_{0}-\frac{4\alpha_{0}^{2}l_{o}}{\pi^{2}}$$

(28) $$\alpha_{02r}=\alpha_{0}-\frac{4\alpha_{0}^{2}(l_{o}+2h+2g)}{\pi^{2}}$$

Compared with the $\alpha_{01}^{2}-\alpha_{02}^{2}$ term, the lift-off height has little effect on the $\alpha_{01}^{2}\alpha_{02}^{2}$ term. Therefore, the $\alpha_{01}^{2}\alpha_{02}^{2}$ term can be considered as a constant *M*.

By substituting $\alpha_{01}$ and $\alpha_{02}$ in (24) with the revised $\alpha_{01r}$ and $\alpha_{01r}$, finally, the liner relationship is obtained, expressed as

(29) $$\tan\theta_{1}-\tan\theta_{2}=\frac{8\omega \sigma \mu_{0}\mu_{r}\alpha_{0}^{3}(g+h)}{M\pi^{4}}*(\pi^{2}-4\alpha_{0}(g+h)-8\alpha_{0}l_{o})$$

Thus, the feature of the difference in the phase tangent of the impedance change is obtained through mathematical derivation, and the effect of lift-off height on this feature is proved to be linear.

## 4. Experiments and Discussions

In this section, we discuss experiments that were conducted to verify the feasibility of the feature of the difference in phase tangent of the impedance change in classification. The impedance change data at different lift-off heights were also collected to verify the linear relationship between the feature and lift-off. In addition, three different excitation frequencies of 40 KHz, 60 KHz, and 80 KHz were used to excite the transmitter coil separately to explore the effect of excitation frequency on this feature and the linear relationship.

### 4.1. Experimental Setup

**Figure 3:**
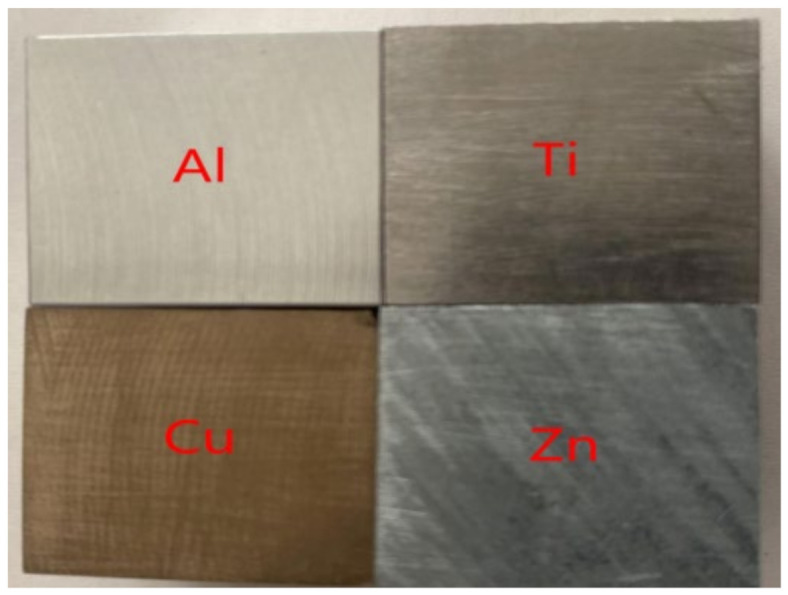
Metals used for the experiment.

**Figure 4:**
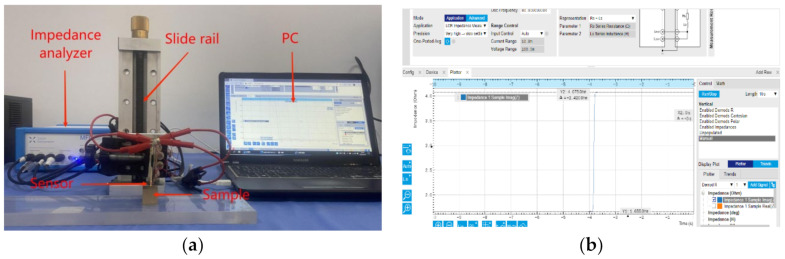
Experimental platform construction. (**a**) System structure; (**b**) measurement page.

In our experiments, four nonmagnetic materials—copper, aluminum, zinc, and titanium, with the geometry of a cube and a side length of 20 mm—were utilized, as shown in Figure 3. The materials’ electrical conductivities are shown in Table 2. These materials are linear and homogeneous. During the experiment, the triple-coil sensor was placed co-axially with the sample under test, with lift-off change assessed only on the vertical axis. The lift-off in the range of 1–5 mm with a step of 1mm was controlled by a slide rail for scale. The impedance change in the two coil pairs was measured using the Zurich impedance analyzer. The experimental platform is shown in Figure 4. Due to the phase difference between the induced voltage and the excitation current, the tested impedance should be complex. The real and imaginary parts of the impedance change can be obtained using the following equation:

(30) $$\Delta Z=Z_{s}-Z_{a}$$

where $Z_{s}$ denotes the impedance of the coil pairs above the sample and $Z_{a}$ denotes the impedance of the coil pairs in free space. $\Delta Z_{1}$ and $\Delta Z_{2}$ denote the impedance change for TR1 and TR2, respectively.

### 4.2. Experimental Results and Analysis

**Figure 5:**
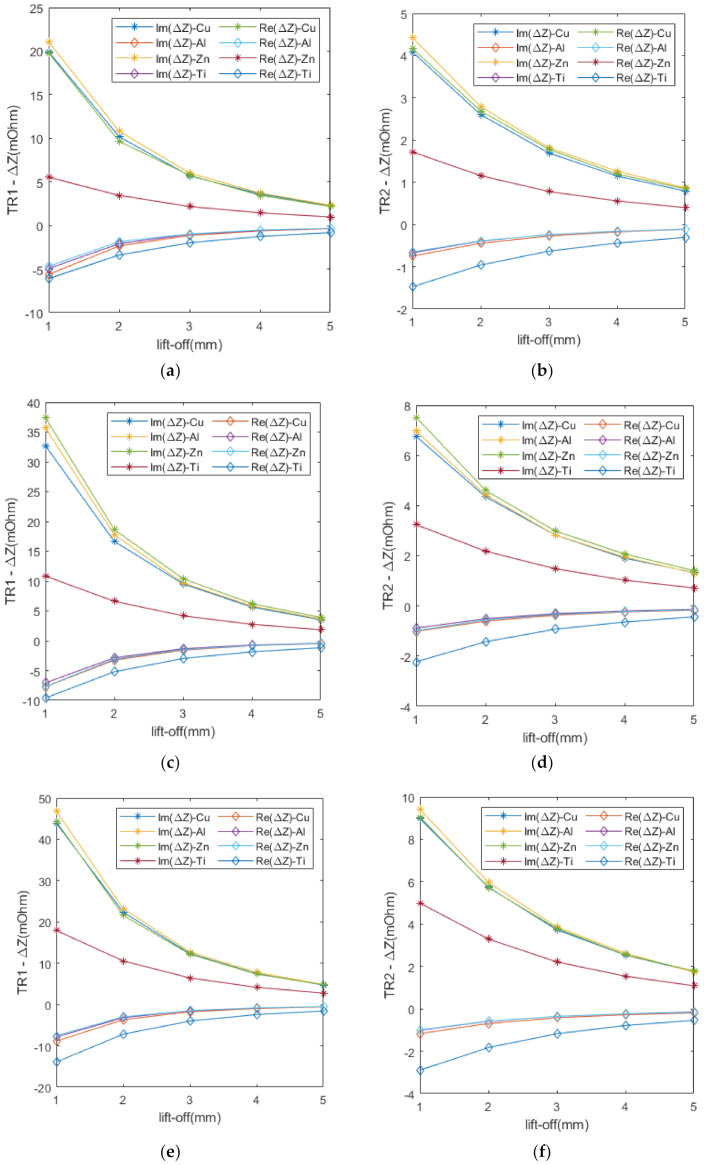
Impedance change in TR1 and TR2 for different metals at different excitation frequencies: (**a**) TR1–40 kHz; (**b**) TR2–40 kHz; (**c**)TR1–60 kHz; (**d**) TR2–60 kHz; (**e**) TR1–80 kHz; (**f**) TR2–80 kHz.

Both the real and imaginary parts of the measured impedance change in TR1 and TR2 at different excitation frequencies are shown in Figure 5. The absolute values of both the real and imaginary parts of the impedance change decay as the lift-off increases. For the same metal, the absolute values of both the real and imaginary parts of the impedance change in TR1 are larger than those of TR2, which is due to the fixed sensor structure of TR2 over TR1, resulting in a smaller lift-off of TR1 than that of TR2. In addition, the absolute values of the real and imaginary parts of the impedance change increase with the increase in the excitation frequency and, in fact, a larger excitation frequency can be utilized to obtain more accurate data when measuring small signals.

**Figure 6:**
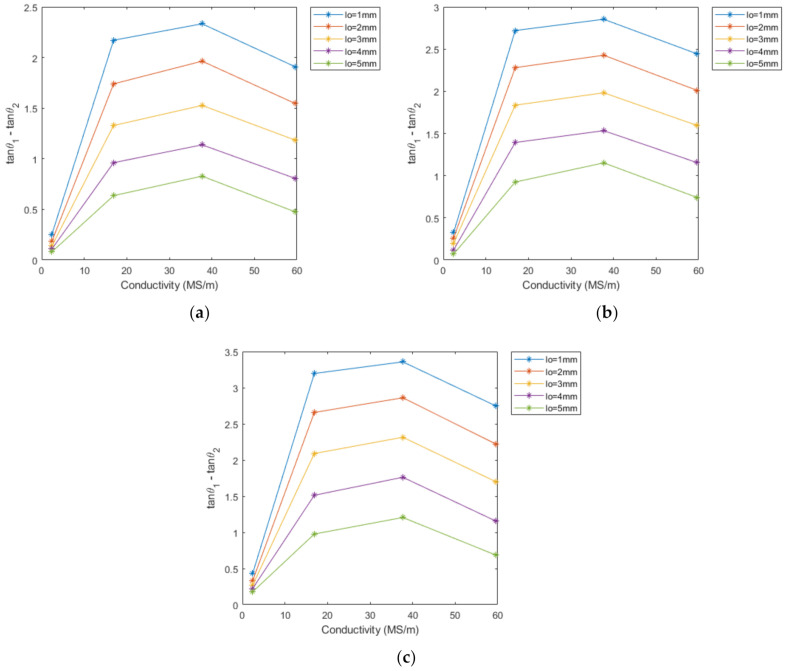
The feature with conductivity as a variable at different lift-offs and excitation frequencies: (**a**) 40 kHz; (**b**) 60 kHz; (**c**) 80 kHz.

Figure 6 illustrates the relationship between the difference in phase tangent and conductivity at different excitation frequencies under the lift-off in the range of 1–5 mm. In this figure, the conductivity refers to the corresponding metal, namely copper, aluminum, zinc, and titanium. The difference in phase tangent can be used as a feature for classification at the same lift-off, since the values of the feature corresponding to the conductivity is different for each metal. The value of the feature decreases with the increase in the lift-off height. Although the magnitude of the difference in phase tangent varies at different excitation frequencies, the trend remains the same when the lift-off varies. According to Figure 6, it is feasible to classify metals at a given lift-off height, while using this feature as the basis for classification is negated when there is lift-off variation. The classification method when the lift-off height varies is explained later in this paper.

Note that the relationship between the difference in phase tangent and conductivity is not exactly proportional, as described in (26). This is due to the material properties described in [28] and [29]. Under the influence of the magnetic field generated by the excitation coil, aluminum, zinc, and titanium exhibit para-magnetism, which means that their relative magnetic permeability is slightly greater than one. Copper, however, exhibits diamagnetism, which means that its relative magnetic permeability is slightly less than one. This is the reason why the feature value of copper is smaller than those of aluminum and zinc. Nevertheless, the difference in phase tangent can still be used as a feature for classification since it can be observed from the figure that the feature values are different for the same lift-off height. 

**Figure 7:**
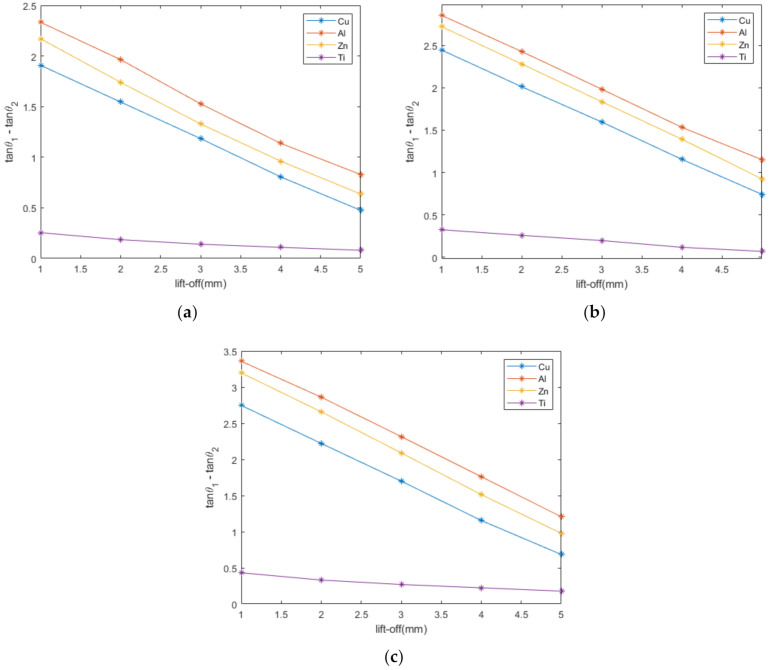
The linearity between the feature and lift-off for different metals at different excitation frequencies: (**a**) 40 kHz; (**b**) 60 kHz; (**c**) 80 kHz.

The linearity of the difference in phase tangent and lift-off is perfectly verified by the experimental data shown in Figure 7. Moreover, the linearity is well maintained regardless of the type of metal and the excitation frequency. The titanium alloy is easily discerned from the plot because its feature value is very different from those of other metals, and it has the smallest slope. According to (29), conductivity does have a slight effect on the slope, but this effect is difficult to observe on the graph, except for the titanium alloy. The reason why the titanium alloy is distinct from the other metals is that its electrical conductivity is very different.

With this linear relationship, conductivity classification can be achieved by this feature even when the lift-off height varies. This will lead to a great improvement in the low classification accuracy caused by the variations in lift-off due to vibrations in the real-time detection classification system.

### 4.3. Classification Method and Accuracy Verification

The method to achieve the classification of conductivity at different lift-off heights is summarized below:

(1) Obtain the impedance change using the triple-coil sensor, and acquire the feature of the difference in the phase tangent of the impedance change after data processing.
(2) Use auxiliary means to measure the lift-off height between the sensor and the sample when measuring the impedance change.
(3) Mark the data point with the lift-off height as the horizontal coordinate and the feature as the vertical coordinate on the feature lift-off plot shown in Figure 7.
(4) Find the line closest to that data point.
(5) Obtain the metal type based on that line.

**Figure 8:**
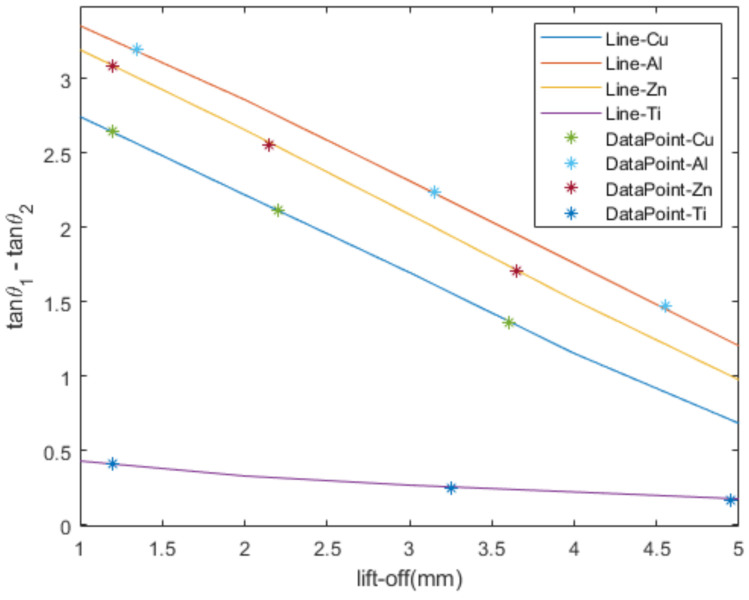
Experimental verification results.

Another set of experiments was conducted to verify the feasibility of the proposed metal classification method when the lift-off height varies, as shown in Figure 8. In this set of experiments, the impedance change in each metal was measured at three lift-off heights, which differed from previous experiments in the range of 1–5 mm. The excitation frequency was 80 KHz.

As can be seen in the figure, the measured data points closely surround the line of the corresponding metal. It is evident that it is feasible to use this method to classify the electrical conductivity of the metals even in the presence of lift-off variation.

## 5. Conclusions

In this study, a classification method based on the eddy current technique is proposed for the classification of nonmagnetic metals. Firstly, we theoretically analyze a physical model of the triple-coil electromagnetic sensor and derive a classification feature related to the sample conductivity. Secondly, the effect of lift-off height on this feature is considered, and it is found that there is a linear relationship between lift-off height and this feature. Thirdly, the feasibility of this feature as a basis for the classification of nonmagnetic metals and the existence of this linear relationship are experimentally verified. Finally, based on this linear relationship, a classification method under the influence of lift-off height is proposed, which greatly improves the problem of low classification accuracy due to vibration-induced lift-off interference in real-time classification systems. In addition, this paper transforms the method of spectrum scanning into the method of single-frequency excitation, which greatly improves the efficiency of classification. As such, the method proposed in this study is very useful for reducing the time cost of large throughput real-time classification systems.

## Figures and Tables

**Table 1:** Coil parameters.

Parameter	Value
Inner radius of the coil (r_1_)	0.8 mm
Outer radius of the coil (r_2_)	1.5 mm
Height of the coil (h)	1.9 mm
Gap between the coils (g)	1 mm
Number of turns (N)	100

**Table 2:** Conductivity of the metals.

Metal	Conductivity (MS/m)
Copper	59.6
Aluminum	37.7
Zinc	16.9
Titanium	2.4

## Data Availability

Not applicable.
